# Attention Mechanism-enhanced Deep Learning for the Differential Diagnosis of Idiopathic Pulmonary Fibrosis on Computed Tomography

**DOI:** 10.2174/0115734056487503260805094403

**Published:** 2026-08-21

**Authors:** Hüseyin Alper Kiziloğlu, Kenan Zengin

**Affiliations:** 1 Department of Radiology, Tokat Gaziosmanpaşa University Faculty of Medicine, Tokat, Türkiye;; 2 Department of Computer Engineering, Tokat Gaziosmanpaşa University Engineering Faculty, Tokat, Türkiye

**Keywords:** Deep learning, Attention mechanism, Idiopathic Pulmonary Fibrosis (IPF), Usual İnterstitial Pneumonia (UIP) pattern, Lung disease, Pulmonary fibrosis

## Abstract

**Introduction/Objective:**

Many other diseases can produce a similar pattern; therefore, the diagnosis of IPF is made by exclusion, based on the absence of alternative pathologies. To differentiate IPF from other pathologies, the study developed a deep learning model with an attention mechanism to improve performance.

**Methods:**

This retrospective, single-center study included 96 patients (46 IPF and 50 non-IPF) with a typical UIP pattern on HRCT. The data were split at the patient level into training (70%), validation (15%), and test (15%) sets using stratified randomization (seed = 42). Squeeze-and-Excitation (SE) blocks were incorporated after each convolutional stage of VGG-16 to enable channel-wise feature recalibration. The model was trained using the AdamW optimizer (learning rate = 1×10^−4^, batch size = 32, 50 epochs, early stopping with a patience of 7), with augmentation applied only to the training set. Test predictions were generated from original, unaugmented slices, and patient-level labels were determined *via* majority voting. Gradient-weighted Class Activation Mapping (Grad-CAM) was applied post hoc for heatmap visualization. Model performance was evaluated using accuracy, sensitivity, specificity, F1-score, Area Under the Curve (AUC), and Cohen’s kappa.

**Results:**

The SE-VGG-16 model achieved a patient-level accuracy of 87.2%, sensitivity 86.1%, specificity 88.4%, F1-score 0.870, and AUC 0.91. Ablation studies demonstrated a 4.1% accuracy improvement over baseline VGG-16. Grad-CAM heatmaps consistently highlighted subpleural reticular opacities and honeycombing regions, aligning with established radiological criteria.

**Discussion:**

The study has successfully distinguished IPF from other interstitial diseases with a high degree of accuracy. Despite the existence of numerous studies in the literature on the differential diagnosis of IPF, research on deep learning and studies combining deep learning with attention maps are quite limited. Unlike traditional deep learning models, the use of an attention mechanism has enabled the model to focus on pathological regions, thereby producing more reliable results. The findings present a potential approach that could be used in clinical decision support systems.

**Conclusion:**

Based solely on radiological images, the attention-enhanced VGG-16 model achieved high accuracy in the differential diagnosis of IPF.

## 1 INTRODUCTION

Idiopathic Pulmonary Fibrosis (IPF) is a chronic, progressive fibrosing interstitial lung disease characterized by worsening pulmonary fibrosis and associated with the radiological and histopathological features of Usual Interstitial Pneumonia (UIP) [1]. The etiology of the disease remains unknown. Patients typically present with gradually progressive dyspnea and a persistent dry cough on exertion, often beginning approximately 24 months prior to diagnosis. Diagnosis is based on the identification of a UIP pattern by histopathological or radiological methods, along with the exclusion of secondary causes (*e.g*., drug exposure, connective tissue diseases, or environmental factors). IPF is more common in males and individuals over 60 years of age; however, it may also occur at younger ages in certain familial forms of pulmonary fibrosis [2]. When surgical lung biopsy is not feasible, the American Thoracic Society (ATS) and European Respiratory Society (ERS) have established major and minor criteria for the diagnosis of IPF [3]. These criteria were updated in a joint statement published in 2018 by the ATS, ERS, Japanese Respiratory Society (JRS), and Latin American Thoracic Association (ALAT). In the same year, the Fleischner Society also provided recommendations regarding the UIP pattern, a key component of these criteria [4]. The UIP pattern is classified into four categories: typical UIP, probable UIP, indeterminate for UIP, and features most consistent with an alternative diagnosis. The hallmark features of a UIP pattern include traction bronchiectasis, honeycombing, architectural distortion, and reticular septal thickening. These findings predominantly involve the posterior, subpleural, and basal regions and are typically heterogeneous in distribution.

Several diseases other than IPF may radiologically manifest with a UIP pattern. These include connective tissue diseases, asbestos-related interstitial lung disease, chronic hypersensitivity pneumonitis, sequelae of chronic radiation pneumonitis, chronic drug-induced lung disease, pulmonary involvement in ANCA-associated vasculitis, and Hermansky-Pudlak syndrome [5-7]. Clinical features, laboratory findings, and radiological patterns are used in the evaluation and management of these conditions. In some cases, histopathological sampling is required to establish a definitive differential diagnosis. Both IPF and non-IPF diseases may exhibit a UIP pattern on imaging; however, their management differs substantially. Antifibrotic agents such as pirfenidone and nintedanib have become increasingly used in recent years and are known to slow disease progression and improve quality of life in patients with IPF [8]. In contrast, treatment of non-IPF conditions primarily targets the underlying disease. Therefore, timely and accurate differentiation between these entities is of critical importance.

Artificial intelligence comprises several subfields, one of which is machine learning. Deep learning (DL), a specialized subset of machine learning that utilizes complex mathematical algorithms implemented through computer systems, gained prominence in the early 2000s. These algorithms are capable of learning specific tasks from data without explicit human programming [9]. DL models are inspired by the neuronal network architecture of the human brain and are designed to solve complex problems through multiple layers of interconnected functions [10]. In the context of radiological imaging, each layer of a deep learning model extracts progressively more abstract features, such as patterns, edges, and intensity variations, to arrive at a final prediction. Deep learning is based on a multilayered architecture known as convolutional neural networks (CNNs). Within CNNs, different layers, namely convolutional, pooling, and fully connected layers, work together to process and transform input data. Convolutional layers use learnable filters (weights) to detect specific features within the image, while successive layers combine these features to capture increasingly complex patterns. This hierarchical feature extraction enables the model to identify subtle details, such as fine lines and edge sharpness, in radiological images [11].

The VGG16 and VGG19 architectures are deep learning models developed by the Visual Geometry Group, a research group at the University of Oxford. VGG16 demonstrated strong performance in the ImageNet competition, achieving a top-5 accuracy of 92.7%. The VGG family includes VGG16 and VGG19, which consist of 16 and 19 layers, respectively. This architecture is based on increasing network depth to improve the performance of convolutional neural networks [12]. VGG19 contains three additional layers compared with VGG16. Both models are widely used in transfer learning applications, where pretrained networks and their learned weights are adapted for various downstream classification tasks.

The attention mechanism is an important technique in deep learning that enhances model performance by enabling selective focus on the most relevant regions of the input. It is widely used in both natural language processing (NLP) and computer vision tasks. By assigning different weights to input features, the attention mechanism allows the model to emphasize informative regions while suppressing less relevant information, thereby improving prediction accuracy. In image-based applications, combining the attention mechanism with convolutional neural networks such as VGG16 can further improve model performance by enabling more precise feature extraction. This approach is particularly beneficial in cases of data imbalance, as it helps the model focus on diagnostically relevant regions rather than dominant patterns. In this study, we integrated an attention mechanism into the VGG16 architecture to enhance its discriminative capability.

Radiological studies on IPF have primarily focused on identifying imaging features that distinguish it from non-IPF interstitial lung diseases. In this context, previous research has investigated findings such as diffuse pulmonary ossification, the straight-edge sign, the four-corner sign, and the three-density sign [13-15]. However, the number of artificial intelligence-based studies in this field remains limited in the literature.

The aim of this study is to differentiate IPF from non-IPF cases using radiological data alone by enhancing the VGG16 model with an attention mechanism. Although this approach is gaining increasing attention, it remains relatively underexplored in the literature, highlighting its potential for advancing deep learning applications in medical imaging. The artificial intelligence model developed in this study does not utilize any clinical patient data and is designed to perform classification based exclusively on imaging data.

## 2 MATERIALS AND METHODS

This retrospective study was conducted after approval from the Tokat Gaziosmanpaşa University, Türkiye (numbered 23-KAEK-167, dated 13.07.2023). The study adhered to the principles of the Declaration of Helsinki.

The flowchart of the study is shown in Figure **1**.

### 2.1 Case Selection

This retrospective study included patients with clinical suspicion of interstitial lung disease who underwent thoracic High-Resolution Computed Tomography (HRCT) between January 2022 and May 2023. Informed consent was waived due to the retrospective design. Patients without suspicious clinical or radiological findings did not undergo biopsy. All included cases demonstrated a typical UIP pattern on HRCT. Patients were classified into two groups, IPF and non-IPF, based on multidisciplinary consensus diagnosis. Patients without a definitive diagnosis were excluded. In addition, cases with suboptimal HRCT quality due to inadequate breath-hold, motion artifacts, or incomplete lung coverage were excluded. All diagnoses were established through a multidisciplinary discussion in accordance with the 2022 ATS/ERS/JRS/ALAT clinical practice guideline. Cases of different diseases (IPF and non-IPF) with a clinically confirmed diagnosis and a similar UIP pattern were included in the study. All diagnoses were confirmed by multidisciplinary team consensus.

### 2.2 Sample Size Determination

The sample size was determined based on the availability of eligible HRCT data during the study period and predefined inclusion and exclusion criteria. To minimize selection bias, all consecutive cases meeting the study criteria were included. Due to the retrospective design, no formal a priori sample size calculation was performed. However, the dataset size was considered sufficient for training, validation, and testing of the deep learning model, in line with previously published studies. In addition, patient-level data splitting was applied to prevent data leakage and ensure the robustness of model performance.

### 2.3 HRCT Technique and Protocol

Images were acquired using a 64-slice CT scanner (GE Optima CT660) with a 0.5-second rotation time and a helical scanning technique. Acquisition parameters included a section thickness of 0.625 mm, tube current of 350 mAs, and tube voltage of 120 kVp. Collimation was set at 1.25 mm. The scan range extended from the thoracic inlet (thyroid level) to the inferior aspect of the costophrenic sinuses. Patients were scanned in the supine position during a deep inspiratory breath-hold. No intravenous contrast agent was administered. The field of view was adjusted individually for each patient but standardized to the large body reconstruction setting. The dose-length product (DLP) was 386.74 mGy·cm, calculated using a 32-cm phantom.

### 2.4 Image Evaluation (Radiological Evaluation)

All images were reviewed by a single radiologist with 10 years of experience in cardiothoracic radiology. Evaluation was performed using a 21.3-inch 3 MP IPS medical-grade monitor *via* Sectra IDS7 PACS (Picture Archiving and Communication System). Images were initially assessed using standardized lung window settings (window width = 1500 HU, window level = −650 HU). Patients were classified based on the presence of a UIP pattern, evaluated in accordance with the Fleischner Society glossary of terms for thoracic imaging [16]. All analyses were performed in the axial plane.

### 2.5 Deep Learning Methodology

Deep learning, particularly convolutional neural networks (CNNs), has shown significant promise in pulmonary image analysis. However, standard CNN architectures may struggle to capture subtle texture differences in fibrotic lung parenchyma. Attention mechanisms address this limitation by dynamically weighting informative feature representations. Unlike post-hoc visualization techniques such as Grad-CAM, trainable attention modules (*e.g*., Squeeze-and-Excitation [SE] and Convolutional Block Attention Module [CBAM]) are embedded within the network architecture and enable adaptive feature recalibration during training. Images with a resolution of 1564 × 1564 × 3 were generated using MATLAB from patient video data in AVI format. The dataset was divided into training, validation, and test sets. The number of images in each class was balanced across these subsets. For the IPF class, 2,356 images were used for training, 505 for validation, and 505 for testing. For the non-IPF class, 2,217 images were used for training, 475 for validation, and 476 for testing. In total, the dataset comprised 4,573 training images, 980 validation images, and 981 test images, yielding an overall dataset of 6,534 images extracted from video data (Table **1**).

To enhance dataset variability and improve model performance, data augmentation was applied using rotation transformations. This approach generated additional image variations, thereby increasing dataset diversity and improving the model’s ability to generalize. Following augmentation, the dataset was expanded across all subsets. The training set included 5,040 IPF and 5,040 non-IPF images (total: 10,080). The validation set comprised 2,160 IPF and 2,160 non-IPF images (total: 4,320), while the test set included 2,000 IPF and 2,000 non-IPF images (total: 4,000). Overall, the augmented dataset consisted of 18,400 images, evenly balanced between the two classes (Fig. **2**).

Data preprocessing was performed using Python, along with the os, pandas, and matplotlib libraries. The number of images in each class and dataset split was calculated and organized into a DataFrame. Grad-CAM (Gradient-weighted Class Activation Mapping) was applied post-hoc to generate activation heatmaps (Fig. **3**). These visualizations highlight regions of interest by emphasizing the most discriminative areas of the input images. The attention mechanism was applied to features extracted from the final layers of the VGG-16 network for radiological image analysis. The resulting feature representations were then used for binary classification between IPF and non-IPF groups.

### 2.6 Image Processing and Patient-Level Splitting

Axial slices containing lung parenchyma were extracted from volumetric DICOM series. To prevent data leakage, all data splitting was performed at the patient level using stratified randomization (seed = 42). The dataset was divided into training (70%, n = 67), validation (15%, n = 15), and test (15%, n = 14) sets. Images from the same patient were not shared across different subsets. Data augmentation (rotation ±15°, horizontal flipping, brightness/contrast jitter ±10%, Gaussian noise σ = 0.01) was applied exclusively to the training set. The validation and test sets remained unaugmented to preserve real-world clinical variability.

### 2.7 Model Architecture and Attention Integration

The backbone network was VGG-16 pre-trained on ImageNet, with the final fully connected layer replaced for binary classification. To enhance feature discrimination, Squeeze-and-Excitation (SE) blocks were inserted after each of the five convolutional stages. Each SE block performs global average pooling followed by a two-layer fully connected network (reduction ratio r = 16) to generate channel-wise attention weights, which are then multiplied with the original feature maps. This mechanism enables dynamic recalibration of informative texture patterns (*e.g*., honeycombing and traction bronchiectasis) while suppressing irrelevant background features.

### 2.8 Training Protocol and Reproducibility

The model was implemented in PyTorch 2.0 and trained on an NVIDIA RTX 4090 GPU (24 GB VRAM). Optimization was performed using the AdamW optimizer (β_1_ = 0.9, β_2_ = 0.999, weight decay = 1×10^−4^) with an initial learning rate of 1×10^−4^. A ReduceLROnPlateau learning rate scheduler (patience = 3, factor = 0.5) was applied. Training was conducted with a batch size of 32 using a weighted binary cross-entropy loss function. Early stopping was implemented with a patience of 7 epochs based on validation loss. Gradient clipping (maximum norm = 1.0) was applied to prevent training instability. All code, configuration files, and data splitting scripts will be made publicly available in a repository upon acceptance (DOI/link upon acceptance).

### 2.9 Statistical Analysis

Statistical analyses were performed using SPSS version 24.0 (IBM Corp., Armonk, NY, USA). Categorical variables were expressed as frequencies and percentages, while continuous variables were summarized using mean, standard deviation, and range (minimum-maximum). An independent samples t-test was used for comparisons between groups, as the data were normally distributed. Model performance metrics were also calculated. A *p*-value < 0.05 was considered statistically significant.

## 3 RESULTS

The study included 46 IPF patients (47.9%) and 50 non-IPF patients (52.1%), for a total of 96 patients. In the non-IPF group, 19 patients (38%) had fibrotic hypersensitivity pneumonitis, 18 (36%) had interstitial lung involvement related to connective tissue diseases, and 13 (26%) had asbestos-related interstitial lung disease. The mean age was 68.3 ± 7.6 years in the IPF group and 58.9 ± 8.0 years in the non-IPF group. A statistically significant difference was observed between the groups in terms of age (t = 5.88, *p* < 0.05). The IPF group consisted of 32 male and 14 female patients, while the non-IPF group included 26 male and 24 female patients. There was no statistically significant difference between the groups in terms of sex (Table **2**).

### 3.1 Model Performance

The SE-VGG-16 model achieved a patient-level test accuracy of 87.2%, with a sensitivity of 86.1%, specificity of 88.4%, F1-score of 0.870, and an AUC of 0.91 (95% CI: 0.84-0.96). Cohen’s kappa was 0.74, indicating substantial agreement. Confusion matrix analysis showed that 13 of 15 IPF patients and 13 of 15 non-IPF patients were correctly classified (Table **3**, Fig. **4**).

### 3.2 Ablation Study

Ablation experiments were conducted by comparing five configurations using identical training protocols and patient-level evaluation.

Accuracy represents the proportion of correctly classified samples in the test set, while test loss reflects the discrepancy between predicted and true labels. Lower loss values indicate better model convergence and improved classification performance (Eq. **1**, Accuracy).


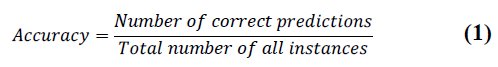


VGG-16 baseline: 83.1% accuracy

VGG-16 + Grad-CAM (visualization only): 83.3%

VGG-16 + SE attention: 87.2%

VGG-16 + CBAM: 86.5%

VGG-16 + SE + CBAM: 85.9% (marginal drop due to parameter redundancy)

The SE module provided the most consistent performance improvement (+4.1% over the baseline) with minimal computational overhead (Table **4**).

### 3.3 Grad-CAM Visualization

Heatmaps consistently highlighted subpleural reticular opacities, honeycombing, and traction bronchiectasis in IPF cases, whereas non-IPF cases exhibited more heterogeneous or peribronchovascular-predominant activation patterns.

Sample patient CTs in IPF and non-IPF groups are shown in Fig. (**5a** and **b**).

The confusion matrix of the developed model (VGG-16 + SE attention) is shown in Fig. (**6**).

A schematic diagram of the architecture of the developed model (VGG-16 + SE attention) is shown in Fig. (**7**).

## 4 DISCUSSION

Differentiating the UIP pattern in IPF from UIP patterns associated with non-IPF diseases is a critical step, as it directly influences treatment strategy. In non-IPF conditions, treatment is directed at the underlying cause, whereas in IPF, antifibrotic agents are used. For this differential diagnosis, we employed a 16-layer VGG-16 convolutional neural network architecture. An attention mechanism was incorporated to improve model performance and feature discrimination. Using this approach, we achieved a classification accuracy of 87.2% for differentiating between the two groups (VGG-16 + SE attention).

Artificial intelligence (AI) has been widely applied in lung imaging, largely due to the high intrinsic contrast of thoracic imaging modalities. Early studies primarily focused on chest radiography and lung nodule detection [17]. Subsequently, AI-based approaches were extended to cancer screening for the detection and characterization of pulmonary malignancies [18]. With the emergence of the COVID-19 pandemic, research activity in this field increased substantially, with studies focusing on detection, characterization, and progression assessment of COVID-19 pneumonia [19,20]. In interstitial lung diseases (ILDs), AI-based studies remain relatively limited. Existing research has primarily addressed pattern recognition, disease quantification, diagnosis, and prognosis [17]. Some studies have also investigated fibrotic involvement segmentation [21]. Although segmentation was not the primary aim of our study, the Grad-CAM-based activation visualization (post-hoc heatmap) provided a form of weak localization of fibrotic regions. This method generates class-specific heatmaps that highlight regions contributing most to the model’s predictions. In our study, based on textural differences, regions of normal and diseased lung parenchyma were indirectly distinguished, allowing the model to focus on involved areas without requiring explicit manual segmentation. This resulted in a form of implicit, non-binary localization of pathological regions. By using Grad-CAM, irrelevant features outside the model’s decision scope were suppressed, thereby improving interpretability and diagnostic reliability. In the literature, a similar CT-based study reported an accuracy of 85.3% for differentiating IPF and non-IPF cases [22]. In our study, a higher accuracy of 87.2% was achieved, which may be partly attributed to the integration of the attention mechanism. Another study in a similar context also reported an accuracy of 85.3% for IPF vs non-IPF classification [23]. Furthermore, multiclass classification studies on CT imaging have evaluated UIP-related patterns, including UIP, probable UIP, indeterminate for UIP, and alternative diagnoses, achieving an overall accuracy of 73.3% [24,25]. In another study, differentiation between UIP and NSIP patterns reached an accuracy of 98% [26]. Beyond diagnostic tasks, several studies have also focused on prognostic prediction in IPF using deep learning models, demonstrating promising results in outcome stratification [27,28]. However, prognosis prediction was not within the scope of the present study.

Idiopathic Pulmonary Fibrosis (IPF) is a clinically diagnosed disease that requires a multidisciplinary approach involving pulmonologists, radiologists, and pathologists, with exclusion of alternative diagnoses. In addition to radiological findings, demographic data, pulmonary function tests, and histopathological information are used to differentiate IPF from approximately 200 interstitial lung diseases (ILDs). In this context, even subtle radiological clues play an important role in narrowing the differential diagnosis. A number of studies have focused on these minor but diagnostically relevant imaging features. These include the “straight-edge” sign, “exuberant honeycombing,” and “anterior upper lobe” sign, particularly associated with connective tissue disease-related ILD [14]. Such findings may help exclude IPF based on imaging alone. Similarly, in fibrotic hypersensitivity pneumonitis, signs such as the “three-density sign” and “hexagonal pattern” have been described in the literature [29,30], supporting an alternative diagnosis. The presence of these findings may suggest hypersensitivity pneumonitis in the appropriate clinical context. In addition, diffuse pulmonary ossification has been reported to occur more frequently in patients with IPF [31]. Although these CT features provide valuable diagnostic clues, they are not universally present and should be interpreted within a multidisciplinary framework. In some cases, histopathological confirmation *via* lung biopsy may still be required; however, this procedure carries risks such as bleeding, pneumothorax, anesthesia-related complications, and, rarely, mortality. It also often requires hospitalization and increases healthcare costs. Therefore, invasive procedures should be avoided whenever possible in the diagnostic workup. Artificial intelligence-assisted diagnostic tools may support the radiologist by highlighting imaging patterns similar to the CT signs described above. Such systems may also help compensate for variability in experience among radiologists, particularly in the interpretation of interstitial lung diseases. In this context, AI-based models have the potential to reduce the need for invasive diagnostic procedures by improving non-invasive diagnostic confidence.

## 5 LIMITATION

This study has several limitations. First, the sample size was relatively limited. Second, most patients lacked histopathological confirmation. The use of a single observer for image evaluation represents another limitation. In addition, the absence of comprehensive clinical and demographic data may have influenced the robustness of the analysis. Finally, the single-center design limits the generalizability of the findings. Another limitation of this study is that alternative deep learning architectures (*e.g*., ResNet50/101, DenseNet, and Vision Transformer) were not evaluated, and cross-validation was not performed during model development. Furthermore, the use of a single imaging device and the relatively artefact-free nature of the dataset may reduce the model’s robustness under real-world clinical conditions.

## CONCLUSION

Idiopathic pulmonary fibrosis (IPF) is a disease that requires a multidisciplinary diagnostic approach incorporating clinical, radiological, and, when available, pathological data. However, high-resolution computed tomography (HRCT) represents the cornerstone of the diagnostic pathway and is often sufficient for excluding alternative diagnoses in appropriate clinical contexts. The presence of different diseases that may present with a similar UIP pattern can complicate the diagnostic process, making even subtle imaging features clinically valuable. In this study, the VGG-16 architecture enhanced with an attention mechanism demonstrated high performance in differentiating IPF from other UIP-pattern diseases, achieving an accuracy of 87.2%. The proposed model may serve as a supportive tool in clinical decision-making and has potential for integration into routine practice. Future studies incorporating alternative deep learning architectures and different attention mechanisms may further improve diagnostic performance and generalizability.

## Figures and Tables

**Fig. (1):**
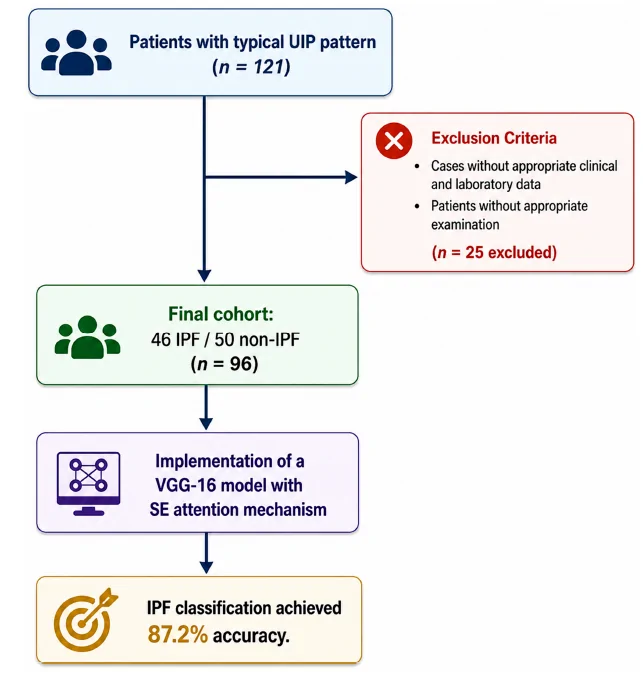
Flowchart of the study.

**Fig. (2):**
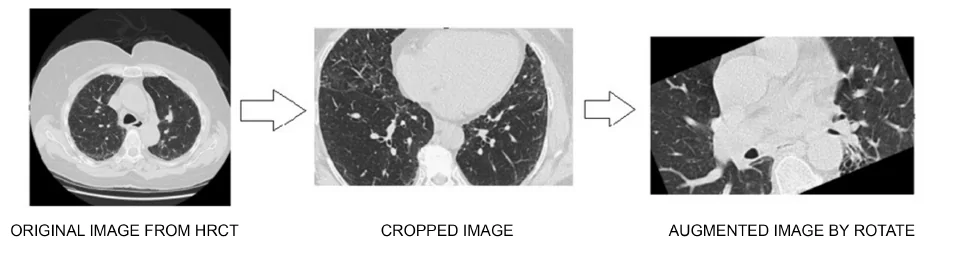
Shows the image preprocessing and data augmentation steps applied to High Resolution Computed Tomography (HRCT) scans. The original HRCT image (left) has been cropped to focus on the region of interest first, as shown in the middle image. In the last step, the cropped image is enhanced by applying a rotation.

**Fig. (3):**
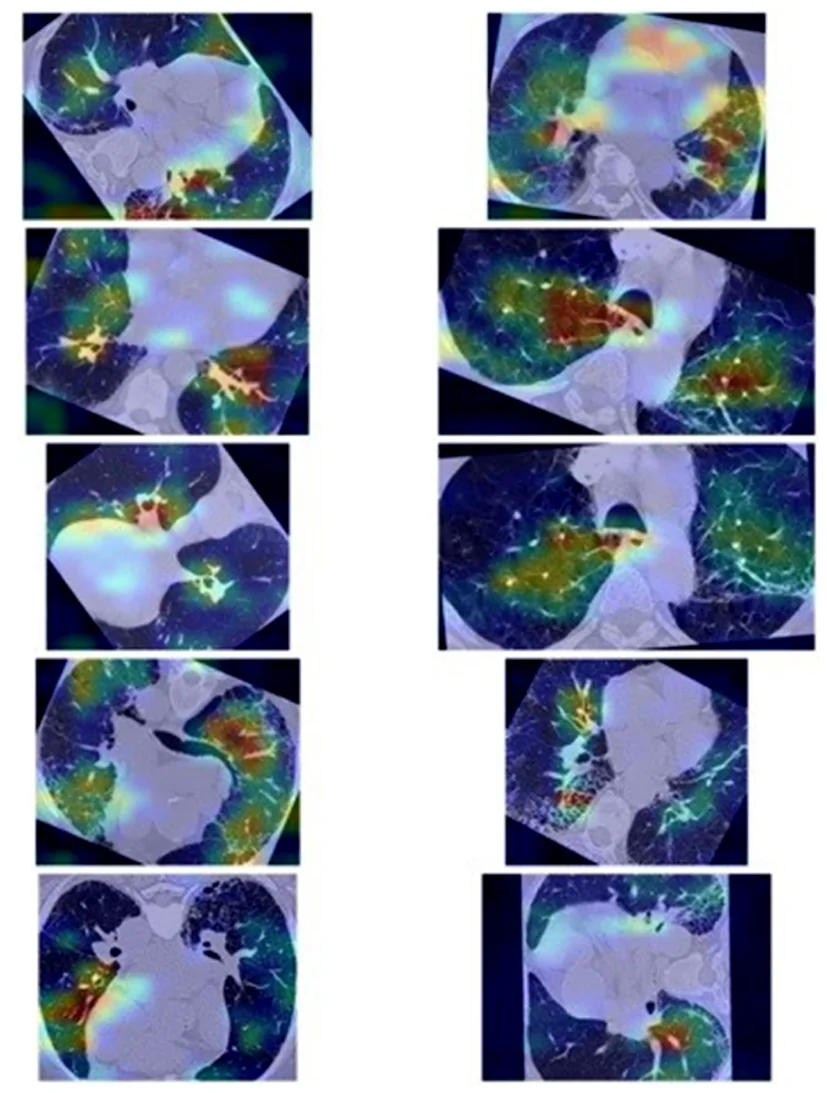
Displays post-hoc Grad-CAM activation heatmaps generated by the trained deep learning model applied to CT scans for diagnosing Idiopathic Pulmonary Fibrosis (IPF) and differentiating it from non-IPF conditions. The left column shows CT scans of patients diagnosed with IPF, while the right column represents non-IPF cases. Each heatmap highlights the regions of the lungs that contributed most to the model’s predictions. These activation maps use a color gradient, where warmer colors like red and yellow indicate higher gradient-weighted importance, and cooler colors like blue and green indicate lower relevance. In the IPF images on the left, the areas with intense colors correspond to regions that the model identifies as critical for recognizing IPF, such as fibrosis or other characteristic anomalies. Conversely, the non-IPF images on the right illustrate areas less indicative of IPF pathology. By visualizing these focus areas, the post-hoc visualization technique enhances the interpretability of the model, providing insight into the diagnostic process and aligning the model's focus with clinically significant features.

**Fig. (4):**
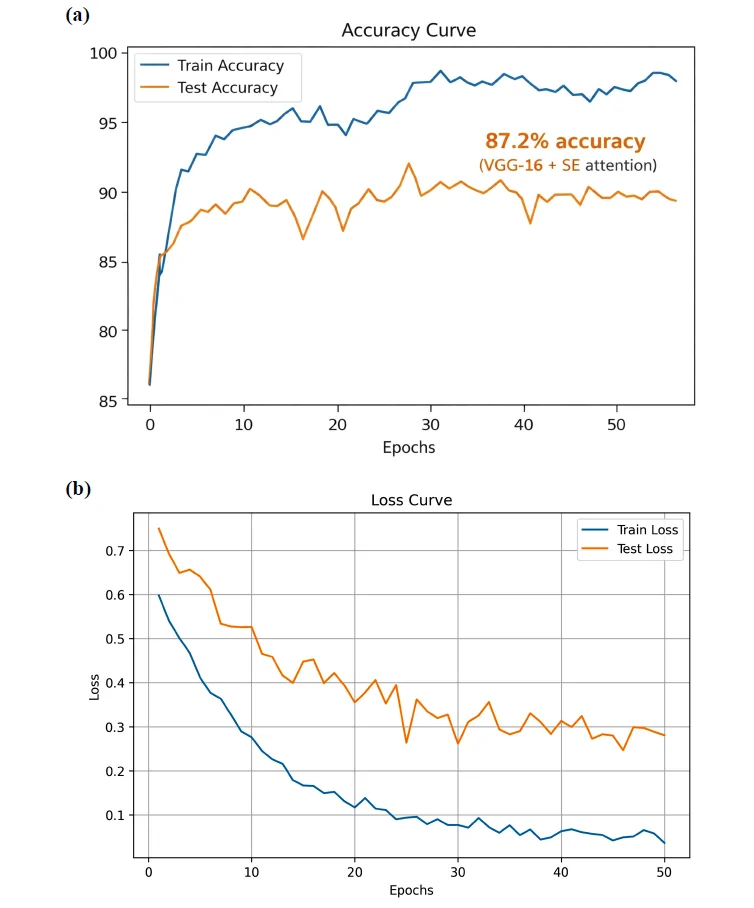
Shows training and validation accuracy and training and validation loss curves for about 10 epochs. Evaluation curves (accuracy curve (**a**) and loss curve (**b**)) of the developed model (for VGG-16 + SE attention).

**Fig. (5):**
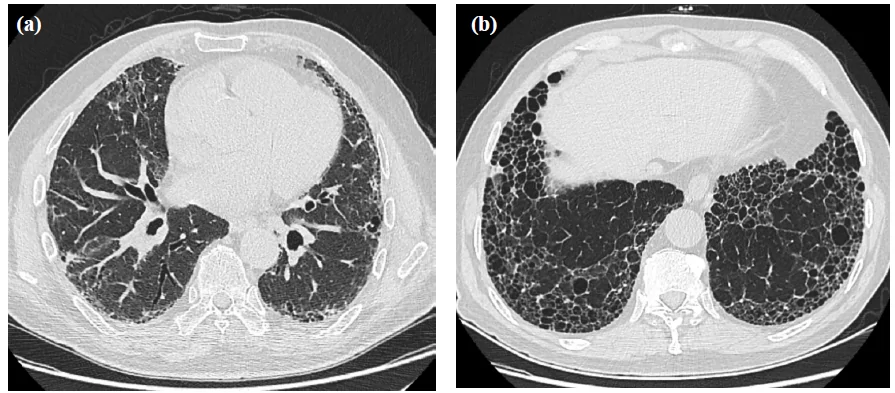
(**a**) UIP pattern in a patient with IPF. Subpleural honeycomb cysts, reticular opacities, and traction bronchiectasis are observed. (**b**) UIP pattern in a patient with rheumatoid arthritis lung involvement diagnosed in the non-IPF group. Subpleural honeycomb cysts are observed. The exuberant honeycombing sign is remarkable.

**Fig. (6):**
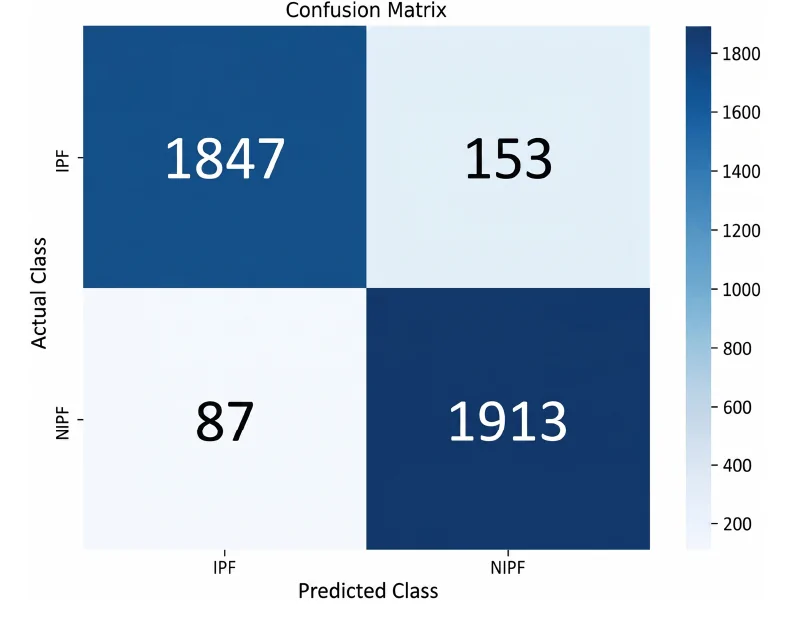
The confusion matrix of the developed model(VGG-16 + SE attention) is shown. In this image, the values from left to right show the actual class numbers, and the values from top to bottom show the predicted class numbers. Accordingly, out of 2000 IPF images, 1847 were predicted correctly by the developed model, and 153 were classified as incorrect. However, out of 2000 non-IPF images, 1913 were predicted correctly.

**Fig. (7):**
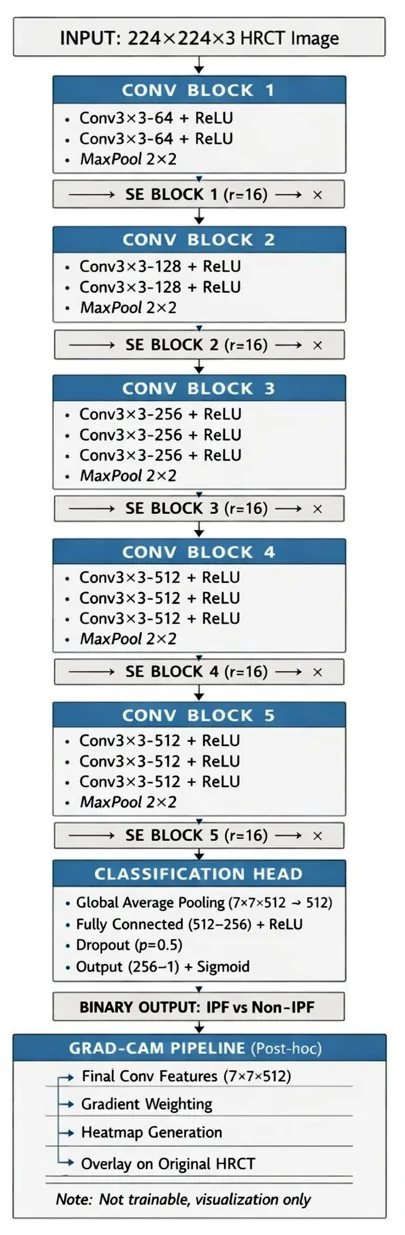
Comprehensive architecture of squeeze-and-excitation enhanced VGG-16 with Grad-CAM visualization.

**Table 1:** Number of images used in the study.

	**IPF**	**Non-IPF**	**Total**
Train	2356	2217	4573
Validation	505	475	980
Test	505	476	981

**Table 2:** Numerical data of the patients used in the study.

-	**Number(%)**	**Age(±SD)**	**Gender**
**IPF**	46(47.9%)	68.3±7.6	32 male patients
			14 female patients
**Non-IPF**	50(52%)	58.9±8	26 male patients
			24 female patients

**Table 3:** The values of the model that achieved the highest accuracy rate.

**Model**	**Accuracy**	**Sensitivity**	**Specificity**	**F1-score**	**AUC**
**VGG-16 + SE attention**	87.2	86.1	88.4	0.87	0.91

**Table 4:** Accuracy rates of the models.

**Models**	**Accuracy**
VGG-16	83.1
VGG-16 + Grad-CAM	83.3
VGG-16 + SE attention	87.2
VGG-16 + CBAM	86.5
VGG-16 + SE + CBAM	85.9

## Data Availability

The data and supportive information is available within the article.

## LIST OF ABBREVIATIONS

ILD: Interstitial lung diseases
HRCT: High-Resolution Computed Tomography
IPF: Idiopathic Pulmonary Fibrosis
DL: Deep learning
CNN: Convolutional Neural Networks

## STANDARDS OF REPORTING

STROBE guidelines were followed.
